# Piloting a Faculty Development Program in a Rural Haitian Teaching Hospital

**DOI:** 10.5334/aogh.3512

**Published:** 2022-03-09

**Authors:** James C. Hudspeth, Nikitha Gangasani, Marc Julmisse, Kerling Israel, Naomie Marcelin, Nadia Raymond, Merly Robert, Zadok Sacks, Christine L. Curry, Michelle Morse

**Affiliations:** 1 Boston University School of Medicine, Department of Internal Medicine, US; 2 Hôpital Universitaire de Mirebalais, Department of Nursing, HT; 3 Private practice, HT; 4 Zanmi Lasante/Partners in Health, Department of Nursing, HT; 5 EqualHealth, US; 6 Hôpital Universitaire de Mirebalais, Department of Internal Medicine, HT; 7 Kaiser Permanente, Department of Obstetrics and Gynecology, US; 8 Harvard Medical School, Department of Global Health and Social Medicine, US

## Abstract

**Background:**

Faculty development for nurse and physician educators has a limited evidence base in high income countries, and very little research from low- and middle-income countries. Health professions educators in many global settings do not receive training on how to educate effectively.

**Objective:**

To pilot and assess a faculty development program aimed at nurse and physician educators at a teaching hospital in rural Haiti.

**Methods:**

We developed a program covering a total of 22 topics in health professions education, including applied learning theory as well as nurse and physician targeted topics. We assessed impact through participant assessment of personal growth, participant evaluation of the program, knowledge testing pre and post program, and structured observations of program participants providing teaching during the program.

**Findings:**

Nineteen out of 37 participants completed the program. While participant reviews were uniformly positive, a pre- and post-test on general educational topics showed no significant change, and the effort to institute observation and feedback of teaching did not succeed.

**Conclusions:**

Our project showcases some benefits of faculty development, while also demonstrating the challenges of instituting faculty development in situations where participants have limited time and resources. We suspect more benefits may emerge as the program evolves to fit the learners and setting.

## Introduction

Human resources for health is a blanket term for the human elements necessary to a functional healthcare system, encapsulating initial training, subsequent deployment, and continuing professional development (CPD) for all health care providers [1,2]. Well delineated paths have developed for the initial education of health care professionals, and while work remains to better match health education with the changing needs of health care systems, the process of training a new nurse or physician performs well when sufficiently resourced [3]. CPD has a more ambiguous track record, with reviews of physician-focused CPD (also termed continuing medical education or CME) finding that many programs fail to change behavior or improve patient outcomes, and that those that succeed at changing behavior often do not sustain this over prolonged periods [4]. Educational programs can struggle to find sufficient time within the busy schedules of health care professionals, and the single-session didactic approach frequently used by CPD programs usually fails at providing lasting changes in knowledge or behavior [5,6].

Low-resource settings amplify the challenges faced by CPD programs. The clinical demands on health care workers are higher, the gaps between provider knowledge and application are wider [7], the logistical and financial support are less [8], and there is scant data to guide program development [9,10], Published programs largely assess via participant satisfaction or immediate knowledge test, while few assess behavior change [11]. Due to the global shortage of health care workers, programs often prioritize faculty development after pressing clinical needs, sacrificing an element key to a quality workforce for pressing short term priorities.

Our research here focuses on faculty development in Haiti, the first Black republic which formed out of a slave rebellion in the 19^th^ century, with subsequent international ostracizing that heavily impeded its growth over the years [12]. Due in part to this, Haiti has a severe shortage of health care workers, with only 14% of the World Health Organization recommended ratio of 4.45 combined doctors, midwives, and nurses per 100,000 population [13]. An unpublished study of Haitian medical education in 2012 found 4 medical school and 33 residency programs across the country; the majority of graduates worked as general practitioners without training beyond medical school (I. Kerling MD, email communication, Jan 2022). A prior survey within Haiti showed poor access to educational opportunities and materials for physicians [14], and graduate medical education programs within Haiti have extremely limited funding to support faculty time and faculty development (M. Morse MD, email communication, April 2018). Nursing schools and medical schools do not routinely provide education on being an effective teacher in either Haiti (I. Kerling MD, email communication, April 2018) or the United States [15].

The Haitian government and the non-governmental organization (NGO) Partners in Health opened a new teaching hospital in March 2013, Hôpital Universitaire de Mirebalais (HUM) [16], staffing it with nursing and medical faculty who largely lacked prior teaching appointments and who often trained in environments deprived of strong educational role models. To address the gap between teaching experience and the desired skills for the new teaching hospital, we designed a pilot CPD program called “Teach the Teacher” to help support the new faculty in their roles as educators. Using Miller’s 4-stage competency assessment model, we aimed to assess knowledge acquisition, self-perceived competence, and demonstration of skills in practice [17].

## Methods

### Course Design

The Teach the Teacher pilot program consisted of didactic and observational components. The didactic portion constituted a series of 22 one-hour lectures and workshops covering core topics for clinical educators in nursing and medicine (see ***Table 1***). Members of the HUM educational leadership and instructors from the NGO EqualHealth arrived at the list of core topics in discussion after review of the literature and existing resources for the faculty development of educators.

**Table 1:** **List of Course Topics** General topics were taught for the entire cohort, with a subset of sessions for specifically physician or nurse participants taught to just those groups.


**General Topics (14)** Orientation and Introduction Adult Learning Theory Curriculum Development Lesson Planning Effective Teaching Techniques Effective Lecturing Designing a Slideshow Facilitating Small Groups Evaluating and Giving Feedback to Trainees Approach to the Problem Trainee Peer Observation of Educators Basic Research Methodology Team-Based Healthcare Introduction to Journal Club	**Physician-Specific Topics (4)** How to Run Teaching Rounds Teaching Clinical Reasoning Core Concepts of Bedside Teaching Effective Clinic Precepting **Nurse-Specific Topics (4)** Leadership in Nursing Mentorship in Nursing Conflict Resolution in Nursing


Literature reviews, expert feedback, and the instructors’ teaching experience were used to develop original context-specific teaching materials. Consistent with best practices in adult education (albeit practices derived outside of the Haitian context) [18], all sessions incorporated active participation and the application of principles by attendees, and typically included interactive lecturing and small group work. Teaching was conducted either in French, the participants’ professional language, or in English with French translation. All slideshows and printed materials were in French.

The course spanned 6 weeks at HUM between December 2014 and February 2016, with 3 topics taught each week in roughly hour-long small group sessions. The majority of topics were meant for both physician and nurse participants, while a smaller number were targeted specifically at a particular profession (see ***Table 1***). The profession-specific topics were chosen by the respective leadership of those professions. Each topic was taught twice during a given week in order to accommodate participants’ clinical schedules.

The observational component involved direct observation of physician participants as they engaged in routine educational activities, specifically while performing teaching rounds, giving feedback to trainees, and providing educational lectures. Activities were evaluated with a rubric providing specific behaviors to be evaluated (Supplemental File 1). Observations were ranked with a score of (1) for not meeting criteria, (2) for needing continued improvement, and (3) for meeting goal criteria. After each observed session, participants were provided with standardized verbal and written feedback. The Teach the Teacher program aimed for each participant to have three or more observations over the program period. Observations occurred both during didactic weeks via visiting teachers and outside of them via project members who held administrative positions at HUM.

### Participants

Select physician and nurse faculty were invited to participate, with HUM leadership determining which faculty were best positioned to use further educational training. We intentionally combined nurses and physicians with intent to build interprofessional connections and model interprofessional education. The pre-established qualifications for a certificate at the end of the course were attendance of half of the sessions, completion of the pre- and post-tests, and undergoing at least three observations of educational activities.

### Monitoring and Evaluation

The program’s impact on participants’ knowledge, attitudes, and teaching practice was evaluated using a multimodal approach. A 29-question multiple-choice test of educational best practices based on course learning objectives was administered at the beginning and end of the program (available as Supplemental File 2), and the comparison was analyzed using Student’s T-test. Qualitative surveys were collected after each individual session and upon the program’s completion to assess participants’ satisfaction with the course and the program’s perceived impact on their professional development. Direct observations of physicians during educational activities were also performed, with a rubric tool to standardize ratings across observers. Our intent was to compare the rubric performance of participants over the course of the year to see whether their individual or communal performance on rubric elements improved.

### Support

The program materials were generated by volunteer nurses and physicians with EqualHealth, and refined in consultation with leaders from HUM. Sessions were largely taught by visiting NGO volunteers. HUM provided room and board for visiting volunteers (normally charging $30 a day), who covered their travel costs to Haiti. EqualHealth covered the approximately $100 necessary for printing course materials and purchasing flash drives containing the course documents for all participants.

### Ethical Review

The study was reviewed by the Zanmi Lasante Research Committee (ZLRC ID#85) and considered to have exempt status as the data generated was produced for monitoring and evaluation of an educational program. We only show program level data throughout this paper to avoid identifying any specific participants.

## Results

Twenty-six physician and eleven nurse HUM faculty members participated in at least two didactic sessions; these thirty-seven faculty members were considered our participants. Fifteen physicians (58% of all physicians) and four nurses (36% of total) participated in at least half of the sessions open to them, which was pre-established as a minimum standard for certification. At least one member of each physician specialty achieved certification at the program’s completion. Nursing representation included two nurse-educators, one rehabilitation nurse and eight nurse managers. The physicians included three participants from emergency medicine, one from rehabilitation medicine, one from family medicine, six from internal medicine, five obstetrician/gynecologists, eight pediatricians and two surgeons.

Thirty-three participants completed the pre-test. The average score from all participants was 57%. Twenty-two participants completed the post-test, with an average score of 63%. The pretest average for nurses was 54% (n = 10) and did not increase for the four nurses who completed the post-test. The physician pre-test average was 59% (n = 23) and increased to 66% (n = 16) (p = 0.08).

The participant evaluation of the program included Likert items related to their role as a teacher. See ***Table 2*** for results from selected individual sessions (research methods, nursing conflict resolution, lesson planning) and from the end-of-course feedback. Not all totals are identical as some participants left responses blank. Across the total of all sessions, 89% of participants agreed with the statement ‘This topic is important to becoming an excellent teacher’ and 87% agreed with that ‘This topic allowed me to better understand my role as a teacher’.

**Table 2:** **Participant evaluation** Participant evaluation of select sessions and summative evaluation from the end of the training.


SESSION: RESEARCH METHODS (N = 29)	AGREE	NEUTRAL	DISAGREE

This topic is important to becoming an excellent teacher	28	1	0

This topic allowed me to better understand my role as a teacher	25	3	0

The teacher demonstrated good understanding of the material	27	1	0

I feel ready to use the material that I learned	23	5	1

Session: Nursing Conflict Resolution (n = 27)			

This topic is important to becoming an excellent teacher	27	0	0

This topic allowed me to better understand my role as a teacher	26	1	0

The teacher demonstrated good understanding of the material	27	0	0

I feel ready to use the material that I learned	26	1	0

Session: Lesson Planning (n = 17)			

This topic is important to becoming an excellent teacher	17	0	0

This topic allowed me to better understand my role as a teacher	17	0	0

The teacher demonstrated good understanding of the material	17	0	0

I feel ready to use the material that I learned	15	2	0

Final Course Feedback (n = 14)			

This course provided me with effective teaching techniques	14	0	0

This course is relevant to my career goals	14	0	0

I am more comfortable working in an interdisciplinary setting because of this course	13	0	0

I feel ready to use the material that I learned	14	0	0


**Figure 1:**
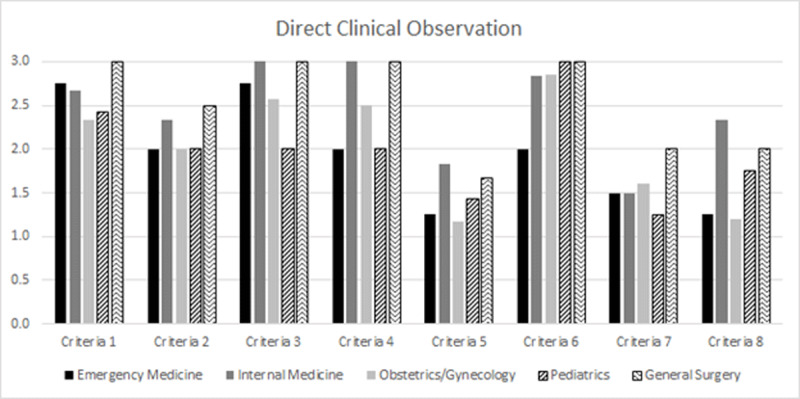
Criteria for evaluation of direct clinical observations (1) attendance and punctuality (2) time management and patient triage (3) leadership and responsibility (4) clinical decision making (5) bedside teaching examination (6) bedside teaching discussion (7) interdisciplinary team approach and (8) integrating patient and families. Observations were ranked with a score of (1) for not meeting criteria (2) needs continued improvement or (3) meets criteria.

There were 27 direct observations of participants conducting multidisciplinary clinical rounds (emergency medicine n = 4, internal medicine n = 6, obstetrics, and gynecology n = 7, pediatrics n = 7, general surgery n = 3), of an intended 78. Across disciplines the lowest score from observers was bedside teaching of patient examination (n = 26, average score 1.46) and the highest score across disciplines was for bedside teaching discussion (n = 26, average score 2.8). The average score for each criterion by department is presented in ***Figure 1***.

## Discussion

We piloted a faculty development program for nurses and physicians at a 300-bed teaching hospital in rural Haiti. While participants broadly lauded the overall program and individual sessions in their survey responses, our tests of knowledge retention did not show statistically significant improvement and our direct observation of teaching returned too few observations to ascertain whether there had been a significant change in behavior. We believe our project provides useful insights into structuring faculty development in a resource-deprived country such as Haiti. There are a range of larger projects that have addressed human resources for health, but many of these focus on training new providers or researchers, and not necessarily on developing the educators [19]. A scattering of US-based programs for training of LMIC educators exist, the most prominent being the FAIMER program, others often being focused on a particular specialty at smaller scale [20,21].

The implemented program had several positive results. We taught an array of topics key to education in nursing and medicine, and the participants routinely viewed the topics as pertinent and well-taught. We additionally assessed behavior change by performing observations of participants teaching, building off of a strong literature on peer observation programs; moving to assessment of behavior change is a potentially important step for faculty development programs globally [22,23], particularly as an intervention that builds communities of educators. We performed this pilot at low cost via the use of volunteer faculty for the design of course materials and actual teaching; while volunteer labor cannot necessarily be scaled up, a country-appropriate curriculum and learning materials can be shared across teaching institutions for use in faculty development once created. We posit that support for country-specific faculty development programs may be a fruitful target for bidirectional partnerships in global health education [24].

Our pilot had several limitations. Due to a short timeline between the decision to develop this program and implementation, the knowledge test was created before the individual session materials were fully completed, and as we received feedback from participants, the focus of what was taught evolved. As such, the final knowledge test did not consistently match the most emphasized teaching points, reducing the utility of our knowledge acquisition test. Since the participants were invited specifically due to their roles as educators, our participants were likely more interested in developing their teaching abilities than the overall faculty and were more likely to favorable rate these activities. Alternatively, as they were asked to join by supervisors, faculty may have felt compelled despite a lack of time or personal interest, which could relate to the overall low rate of completion. We did not survey those who did not complete the program for their barriers to completion, and this would have been useful information. Participants reported considerable limitations on their time between their direct clinical responsibilities, their time teaching trainees, and the administrative roles that most possessed. There was no protected time for these sessions, as distinct from much of the CPD these staff are exposed to, which occur as full-day events, often off site. This lack of time and support, familiar to health professional faculty throughout the world, often precluded faculty attendance, even when offering each session twice in a given week. Of note, the nursing staff reported more time pressure, which may relate to why fewer of them completed the program. Finally, the majority of the sessions were taught in English with French translation; this likely reduced the effectiveness of the education, especially given the complexity of the topics.

The observation portion of the program designed to assess behavior change had two particular challenges. First, direct observation of faculty was novel to this environment, and ultimately few faculty were eager to undergo peer feedback. This may reflect a failure to successfully establish a safe learning environment, a foundational requirement for such programs, or insufficient time exploring this approach with faculty prior to implementing it given its novelty. Second, our peer observers were largely educational administrators for the hospital; this proved a scheduling challenge, as they were quite busy with existent educational, clinical, and administrative duties. Consequently, while we did obtain a number of observations, we fell short of our goal of having sufficient observations of each faculty member to assess for changes in their behavior.

In summary, we implemented a pilot faculty development program in an academic Haitian hospital. Our sessions were well received by the nursing and physician participants, and the course curriculum generated was viewed by participants as relevant to their clinical educator roles. We met challenges in evaluating the program using standard pre and post-testing strategies and in retention of faculty through the entire program. The attempted introduction of direct observation of education highlighted the tension between existing responsibilities and adequate time for faculty development, as well as the challenges of implementing new educational approaches that involve critique of faculty. This program continues as a CPD activity organized by HUM leadership, with reported particular interest from the younger faculty. Health professional educator development in LMICs is an understudied topic that should have downstream benefits for a range of health system metrics and is a space where international partnerships and NGOs can play a useful role supporting LMIC institutions.

## Additional Files

The additional files for this article can be found as follows:

10.5334/aogh.3512.s1 Supplementary File 1. Rounds Evaluation Rubric in French and English.

10.5334/aogh.3512.s2 Supplementary File 2. Pre and post test in French and English.
