# Supplementary material for: Piloting a Faculty Development Program in a Rural Haitian Teaching Hospital
Source: Ann Glob Health. 2022 Mar 9;88(1):19. doi: 10.5334/aogh.3512 (PMC8916063; doi:10.5334/aogh.3512)
Supplement: Supplementary File 1. — Rounds Evaluation Rubric in French and English. [file agh-88-1-3512-s1.pdf]

Physicians for Haiti: Teach the Teacher
Name of Evaluator____________________ Rounding Team Leader Name________________________
Hospital Service______________________ Date_____________________

Criteria for Evaluation	1: Not Meeting Criteria	2. Needs Continued Improvement	3: Excellent
**1. Attendance and Punctuality**	Rounds were not started on time. The attending physician, residents or nurses were not on time.	Rounds were not started on time. Some of the team members but not all were late.	Rounds started on time. All team members arrived on time.
**2. Time Management and Patient Triage**	Some sick or complex patients patients were not seen or discussed.	There was some discussion by doctors and nurses about which patients were sickest or most complex.	There was a clear plan to see the sickest or most complex patients first if they were unstable or needed attention first.
**3. Leadership and Responsibility**	It was not clear who was in charge of rounds. No one took ownership or responsibility for patients.	It was clear who was in charge of rounds. Some of the team members took responsibility for some of the patients.	It was clear who was in charge of rounds and which physician and which nurse was responsible for each patient.
**4. Clinical Decision Making**	No explanation is given for plans or decisions.	Some explanations for decisions are given. Decisions sometimes based on best practices, protocols or evidence.	Leader of rounds consistently states explanations for decisions and can cite best practices, protocols or evidence to support.
**5. Bedside Teaching Examination**	Patients are never or rarely examined.	Physical exam findings of teaching value are pointed out to team by leader and all team members examine. Some discussion of the importance of that finding.	When a physical exam finding is of teaching value the leader of rounds assists all team members in exam and discussion of importance of that finding.
**6. Bedside Teaching Discussion**	Team members are not asked questions on rounds.	Team members are sometimes asked questions that test decision making and fund of knowledge.	Team members are frequently asked questions that test decision making, fund of knowledge, protocols and evidence.
**7. Interdisciplinary Team Approach**	No attempt is made to reach out to nurses or other non-physicians on rounds.	Nurses or non-physicians are involved in the discussion for most but not all patients during rounds.	Nurses or non-physicians are routinely encouraged to discuss patient management.
**8. Integrating Patient and Families**	No attempt is made to include the patient and family in discussion	Patients and families are sometimes involved during rounds	All patients and families are encouraged to participate in rounds

Comments:

Médecins pour Haïti: Formation des Formateurs
Nom de l´évaluateur___________________ Nom du leader de l'équipe de tournée _______________________
Service hospitalier _____________________ Date____________________

Évaluation de la tournée D'enseignement

Critères d’évaluation	1: Ne répond pas aux critères	2. Necessite une amélioration continue	3: Excellent
**1. Assistance et ponctualité**	La tournée n'a pas commencé à l'heure. Le médecin, les résidents ou les infirmiers traitants ne sont pas arrivés à l'heure.	La tournée n'a pas commencé à l'heure. Quelques membres de l'équipe etaient en retard, mais pas tous.	La tournée a commencé à l'heure. Tous les membres de l'équipe sont arrivés à l'heure.
**2. Gestion du temps et triage des patients**	On n'a pas visité ou discuté de quelques patients tres malades ou complexes.	Il y a eu un peu de discussion sur quels patients étaient les plus malades ou complexes.	Il y avait un plan clair pour discuter d’abord des patients les plus malades ou complexes, s'ils n'étaient pas stables ou s'il leur fallait une attention particuliere
**3. Leadership et responsabilité**	Il n'était pas clair qui était le responsable de la tournée. Personne n'a pris la charge ou la responsabilité des patients.	Il était clair qui était le responsable de la tournée. Quelques uns des membres de l'équipe ont pris la responsabilité de quelques patients.	Il était clair qui était le responsable de la tournée et quel médecin et quel infirmier étaient responsables de chaque patient.
**4. Prise de décisions cliniques**	On n'a donné aucune explication pour les plans ou les décisions.	On a donné quelques explications pour les décisions. Les décisions étaient basées parfois sur les meilleurs pratiques, protocoles, ou évidence.	Le leader de la tournée a précisé constamment les explications sur les decisions prises et fait référence aux meilleurs pratiques, protocoles ou évidences pour les soutenir.
**5. Enseignement de l’examen physique au chevet du patient**	Les patients ne sont jamais ou sont rarement examinés.	Les trouvailles de l’examen physique de valeur pedagogique ont ete soulignees par le leader, et tous les membres ont examine. Il y a eu un peu de discussion sur l'importance de ces trouvailles.	Quand une trouvaille d'un examen physique est de valeur pédagogique le leader de la tournée assiste les membres de l'équipe dans l'examen et la discussion se porte sur l'importance de cette trouvaille.
**6. Pedagogie au chevet du patient**	On n'a pas pose de questions aux membres de l'équipe pendant la tournée.	On a pose quelques questions aux membres de l'équipe qui testent leur prise de décision et connaissances.	On a pose des questions aux membres de l'équipe qui testent leur prise de décision, leurs connaissances, les protocoles et l'évidence.
**7. Approche interdisciplinaire et d'équipe**	On n'a pas essayé d’integrer infirmiers et para-medics	Les infirmiers et para-medics ont été inclus dans la discussion pour la plupart des patients durant la tournee	Les infirmiers et para-medics ont été constamment encouragés à discuter de la gestion du patient.
**8. Intégration des patients et de leur famille**	On n'a pas essayé d'inclure le patient et sa famille dans la discussion.	Les patients et leur famille sont parfois inclus dans la tournée.	Tous les patients et toutes les familles sont encouragés à participer dans la tournée.

Commentaires:

Domaines a améliorer:
