# Supplementary material for: Piloting a Faculty Development Program in a Rural Haitian Teaching Hospital
Source: Ann Glob Health. 2022 Mar 9;88(1):19. doi: 10.5334/aogh.3512 (PMC8916063; doi:10.5334/aogh.3512)
Supplement: Supplementary File 2. — Pre and post test in French and English. [file agh-88-1-3512-s2.pdf]

English version of pre/post test – the section titles were removed, and the bolded answer is the correct one. The French translation is appended after the English version.

Adult Learning Theory

Which of these statements about adult learning is TRUE?

a. Adults learn best when they believe that there will be serious consequences for them if they do not learn

b. **Adults learn best when they have the opportunity to observe others modeling activities**

c. Adults always learn best from Powerpoint slides

d. Adults learn best from lectures that are not interactive

According to the principles of adult learning theory, which of the following is NOT an ideal teaching behavior?

a) A teacher asks students to identify their individual learning goals at the beginning of a rotation
b) A teacher provides students with a list of his objectives and goals at the beginning of a lecture
c) While giving a lecture, the teacher refers back to material she discussed with students previously
d) A teacher criticizes a student in front of a group of their peers for not knowing an answer

Effective Teaching Techniques

Which of the following represents an effective adult teaching technique?

a. A teacher conducts a three-hour lesson without any breaks, since learning is more effective if it happens without any interruption

b. **A teacher incorporates an activity into a lesson where students break into small discussion groups**

c. A teacher asks only very specific questions that test students' knowledge

d. A teacher provides their students with long lists of facts for them to copy into their notes

Which of the following is recommended when a teacher is leading a one-hour discussion session with 10 students?

a. The teacher should do almost all of the talking during the session

b. **The teacher should encourage students to challenge the teacher's arguments when they disagree**

c. The teacher begin the session with a 40-minute lecture, then ask if students have any questions

d. The teacher should allow students unlimited time for each comment or question

Curriculum Design

What should be the first step when one is designing a new curriculum for a residency program?

a. Set goals and objectives for the curriculum

b. **Perform a general assessment to understand the program's needs**
c. Identify educational strategies that are well-suited for the material
d. Set dates and times for all learning sessions in the curriculum

Which of the following is considered an effective technique when developing a new educational program for a hospital?

a. **All steps should be considered with the desired end result in mind**
b. The program should be based mainly on the opinions of experts, not those of the people who will be participating
c. Always use techniques that have been shown to be effective in hospitals in the US
d. One should never alter the goal of a program once they have established it

Lesson Planning

Which of the following should a teacher do when planning a one-hour lesson?

a. The teacher should not practice a lecture ahead of time, since it will seem rehearsed
b. The teacher should plan to give numerous examples for each point they make
c. **The teacher should plan to begin a lecture with a brief explanation of why the topic is important**
d. The teacher should plan to fill the entire hour with lecture material

Which of the following represents an "agenda item" rather than a "learning objective"?

a. "Understand key differences between generalized and partial seizures"
b. "Learn two different classification systems for anemia"
c. "Become comfortable with using ultrasound to diagnose ascites"
d. **"Discuss the definition of stroke"**

Effective Lecturing / Ideal Powerpoints

Which of the following is a common error made when giving a lecture presentation?

a) Having too much text on each slide
b) Practicing before you give the presentation
c) Explaining why the topic is important early in the presentation
d) Leaving too much time for questions at the end of the presentation

About how many minutes should you spend on each slide when giving a presentation describing complex concepts?

a) 1 minute
b) 2 minutes
c) 5 minutes
d) 10 minutes

Which of these is the most effective method for teaching using a Powerpoint slide?

A) Putting a picture on a slide, with words summarizing key points, and verbally repeating those points

B) **Putting a picture on a slide, with no words, and verbally describing the processes shown in the picture using plain language**

C) Putting just words on a slide without pictures, and talking through the words

D) Putting a picture on a slide, with words summarizing key points, and verbally describing the processes shown in the picture

Which of the following DOES NOT need to be in a slide show?

a) Title
b) Learning objectives
c) Animations
d) Summary slide

Evaluation and Feedback

Which statement is NOT a characteristic of feedback?

a. Presents information, includes specific details and examples

b. Focuses on actions and behaviors

c. Presents summary judgment and generalizations

d. Should happen throughout the learning process and help keep someone on track

Which of these is a statement of evaluation?

a) Your lecture yesterday was well-organized, but should be more focused on the disease and less on treatments
b) You have done well during this rotation with me
c) I noticed that your dressings for the wounds are not well secured to the patient
d) You have been consistently on time for work, and I appreciate it

Small Group Education

When using small groups for a group learning activity, it is important to assign clear roles for all group members before the small group work begins.

True or False

Small groups are less effective because they do not allow you to evaluate all learners participating in the group learning activity.

True or **False**

Effective Evaluation

Which of the following best summarizes the use of milestone based evaluation compared to traditional evaluations?

a) Milestones allow reviewers to rate a trainee along a scale of 1 to 5 on how well they are doing at their skills
b) Milestones tell a program when a trainee has learned enough to safely progress to independent practice
c) Milestones provide an objective measure of accomplishment based on what trainees can do
d) Milestones measure a trainee's level of knowledge

When developing a system of evaluations for a program, which of the following is usually the most important type of evaluation?

a) Tests of knowledge looking to see how much the trainee has learned
b) Observation of trainee by faculty who compare the trainee performance to program goals
c) Reviews from the peers of the trainee who work alongside them
d) Self-evaluations, where a trainee discusses how they feel they are performing

Peer Evaluation of Educators

What do peer evaluation techniques aim to accomplish for a program?

a) Provide a way for faculty to improve as educators via feedback from fellow faculty

b) Allows program directors to obtain input on how educators are doing based on feedback from fellow faculty
c) Provide a way for faculty to improve as educators via feedback from their directors, who are comparing them to other faculty
d) Provide a way for faculty to improve as educators via feedback from their trainees

Which of the following is an example of a method of peer evaluation?

a) Comparing the scores of different faculty to one another
b) Administrators grading different faculty based on patient outcomes
c) Having another faculty member observe your teaching and then give you feedback on your teaching technique
d) Asking trainees how they would rate different faculty as teachers

Team-based Healthcare (Nurses and Doctors Working Together)

Which of the following are benefits from optimizing coordination between nursing and physician staff?

a) Improved ability for nurses to alert physicians to changes in patient condition
b) Better understanding of physician rationale for nurses, leading to improved ability to carry out orders
c) Decreased conflict between professions due to better communication
d) All of the above

Principles of Professionalism

Which of the following are a pair of values that health care professionals must balance in their treatment of patients?

a) Risks versus benefits
b) Patient autonomy versus authority of the health care professional
c) Beneficence versus maleficence
d) Informing the patient versus keeping the patient ignorant

Which of these sentences best defines professionalism within healthcare?

a) Prioritizing the education of colleagues in your profession above all else
b) Prioritizing increased reimbursement for your profession whenever possible
c) Prioritizing communication with your patients
d) Prioritizing the needs of the patient and society above all else

Developing Mentors

Which of the following best describes the role of a mentor?

a) Someone who is an example of what you want to be by later in your career
b) Someone who provides you with education about areas in your field and assists you with thinking about new projects
c) Someone who helps you think through the opportunities available and helps create opportunities for you
d) Someone who oversees your work and gives you feedback on how you are doing

Which of the following is true of mentorship during a career?

a) We often will have multiple mentors for different aspects of our career
b) The mentors you start with should be your mentors throughout your career
c) The best mentors are usually those superior to you in your own institution
d) A mentor must be someone you meet with very regularly

Incorporating Research into a Career

What type of research is usually the easiest to do?

a) Randomized controlled studies
b) Prospective observational trials
c) Retrospective observational trials
d) Meta-analysis trials

What is the first step in designing a research project?

a) Considering the resources you have available for the project
b) Having a specific question to answer with the project
c) Doing a review of the literature pertinent to the project
d) Submitting forms to the Institutional Review Board

Professional Development

What is most important step in planning your personal development as a professional?

a) Going over what positions you are eligible to be promoted to at your present institution
b) Thinking about how much money you need to have
c) Considering the job you would like to have in several years, then thinking about what steps are necessary to get there
d) Thinking about what research projects would help further your career

What is the rate of dissatisfaction with job-life balance (or burn out) among US nurses and doctors?

a) 10%
b) 15%
c) 25%
d) 40%

Examen de connaissances

Nombre de participant :

1. Parmi les déclarations suivantes sur la pédagogie des adultes, laquelle est vraie?

a. Les adultes apprennent mieux lorsqu'ils pensent qu'il y aura des conséquences à leur manque de connaissances
b. Les adultes apprennent mieux lorsqu'ils ont l'opportunité d'observer d'autres personnes effectuer les activités enseignées
c. Les adultes apprennent mieux avec des présentations Powerpoint.
d. Les adultes apprennent mieux lorsque les cours ne sont pas interactifs.

2. Laquelle des choses suivantes est une erreur souvent commise lors d'une présentation?

a. Avoir trop de texte par diapositive
b. S'entrainer avant de donner la présentation
c. Expliquer pourquoi le sujet est important au début de la présentation
d. Laisser trop de temps pour les questions à l'issue de la présentation

3. Parmi les techniques suivantes, laquelle est efficace lors du développement d'un nouveau programme éducatif pour un hôpital?

a. Toutes les étapes doivent être considérées avec la fin en tête.
b. Le programme doit se baser uniquement sur l'opinion d'experts et pas sur l'opinion des participants.
c. Seulement utiliser des techniques qui se sont prouvées efficaces aux Etats-Unis.
d. Ne jamais changer les objectifs du programme une fois qu'ils ont été établis.

4. Parmi les techniques suivantes, laquelle est une technique de pédagogie adulte efficace?

a. Un professeur fait un cours de trois heures, sans prendre de pause, car les élèves apprennent mieux quand le cours est sans interruption.
b. Un professeur incorpore une activité en binômes ou en petit groupe lors de son cours.
c. Un professeur pose uniquement des questions très spécifiques afin de tester les connaissances de ses élèves.
d. Un professeur fournit à ses élèves une longue liste de faits et de détails à recopier dans leur cours.

5. Lorsque vous formez un groupe pour une activité d'apprentissage, il est important d'assigner des rôles clairs à tous les membres du groupe avant que le travail de groupe ne commence.

Vrai ou Faux

6. Laquelle de ces propositions résume le mieux l'usage du contrôle continu comparé à l'évaluation traditionnelle?

a. Les étapes du contrôle continu permettent aux examinateurs de noter les étudiants de 1 à 5 sur leurs compétences
b. Les étapes du contrôle continu permettent de déterminer le moment où un étudiant a assez progressé pour pouvoir progresser de manière indépendante
c. Les étapes fournissent un objectif mesuré de l'accomplissement basé sur les capacités de l'étudiant
d. Les étapes mesurent le degré de connaissance d'un étudiant

7. Laquelle de ces propositions les professionnels de la santé doivent-ils équilibrer dans leur traitement des patients ?

a. Risques contre bénéfices
b. Autonomie du patient contre autorité du professionnel de santé
c. Bienfaisance contre non-malfaisance
d. Informer le patient contre le fait de ne pas informer le patient

8. Laquelle de ces propositions s'applique au mentorat lors d'une carrière ?

a. Nous aurons souvent différents mentors pour différents aspects de notre carrière
b. Les mentors avec lesquels nous commençons notre carrière devraient être nos mentors tout au long de notre carrière
c. Les meilleurs mentors sont souvent vos supérieurs dans votre institution
d. Un mentor est une personne que vous devew rencontrer régulièrement

9. Approximativement combien de minutes par diapositive doit-on prendre pour expliquer un concept complexe?

a. 1 minute
b. 2 minutes
c. 5 minutes
d. 10 minutes

10. Lorsque vous développez un système d'évaluations pour un programme, laquelle de ces propositions est généralement la plus importante?

a. Les tests de connaissance permettant de déterminer l'avancée de l'apprentissage d'un étudiant
b. L'observation d'un étudiant par un professeur qui compare ses perfomances aux objectifs du programme
c. Revue par les pairs d'un étudiant
d. L'auto-évaluation par laquelle un étudiant peut discuter de son degré de performance

11. Les petits groupes sont moins efficaces car ils ne permettent pas d'évaluer tous les participants participant à l'activité de groupe.

Vrai ou Faux

12. Que permet l'évaluation par les pairs dans l'accomplissement d'un programme?

a. Permettre à un professeur d'améliorer ses compétences d'éducateur grâce au feedback d'un autre professeur
b. Cela permet aux directeurs de programme d'obtenir des conseils sur la manière dont les éducateurs travaillent grâce au feedback des autres directeurs
c. Permettre à un professeur d'améliorer ses compétences grâce au feedback des directeurs qui les comparent aux autres professeurs
d. Permettre aux éducateurs d'améliorer leurs compétences grâce au feedback de leurs étudiants

13. Selon les principes de la pédagogie des adultes, laquelle des techniques d'enseignement suivantes n'est pas idéale?

a. Un professeur demande à son élève d'identifier les objectifs de son éducation au début d'un stage.
b. Un professeur fournit à ses élèves une liste d'objectifs au début de son cours.
c. Lors de son cours, le professeur fait référence aux cours précédemment discutés avec les élèves.
d. Un professeur critique un(e) élève devant ses pairs lorsqu'il/elle ne connait pas la réponse

14. Parmi les choses suivantes, lesquelles doivent faire un professeur lors de la planification d'un cours d'une heure?

a. Le professeur ne doit pas répéter son cours à l'avance sinon il aura l'air trop chorégraphié.
b. Le professeur doit préparer de nombreux exemples pour chaque élément de cours.
c. Le professeur doit se préparer à commencer son cours avec une explication de pourquoi son cours est important.
d. Le professeur doit être préparer à remplir l'heure entière avec son cours.

15. Parmi les options suivantes, laquelle est recommandée lors d'un cours où le professeur mène une discussion d'une heure avec 10 étudiants?

a. Le professeur doit parler la majorité de l'heure
b. Le professeur doit encourager les élèves à questionner ses arguments lorsqu'ils sont en désaccord.
c. Le professeur débute l'heure avec une discussion de 40 minutes, puis demande aux élèves s'ils ont des questions.
d. Le professeur doit laisser aux élèves un temps illimité pour chaque question ou commentaire

16. Quel type de recherche est généralement le plus simple à faire ?

a. Des études cliniques aléatoires
b. Des essais cliniques prospectifs
c. Des essais cliniques rétrospectifs
d. Des essays méta-analytiques

17. Qu'est-ce qui est le plus important pour votre développement personnel en tant que professionnel ?

a. Surveiller les postes pour lesquels vous pourriez être promu
b. Calculer le salaire que vous souhaiteriez avoir
c. Imaginer le poste que vous souhaiteriez avoir dans quelques années et ensuite penser aux étapes nécessaires pour y parvenir
d. Déterminer les projets de recherche qui peuvent vous aider à faire avancer votre carrière

18. Quel est le taux de dissatisfaction entre l'équilibre travail-vie sociale (ou burn out) pour les médecins et infirmières des Etats-Unis ?

a. 10%
b. 15%
c. 25%
d. 40%

19. Laquelle des choses suivantes n'est pas essentielle à une présentation Powerpoint?

a. Un titre
b. Des objectifs éducatifs
c. Des animations
d. Une diapositive résumant la présentation

20. Parmi les critères suivants, lequel n'est pas caractéristique de commentaires constructifs?

a. Présente l'information et inclue des détails et exemples précis
b. Se focalise sur les actions et les comportements
c. Présente un jugement sommaire et des généralisations
d. Doit se faire tout au long du processus éducatif, de manière longitudinale, afin de maintenir l'élève dans un bon chemin

21. Laquelle des ces propositions décrit le mieux le rôle d'un mentor ?

a. Une personne dont l'exemple vous inspire pour la suite de votre carrière
b. Une personne qui vous éduque dans votre domaine et vous assiste dans le développement de nouveaux projets
c. Une personne qui vous aide à réfléchir aux possibilités disponibles et vous aide à vous créer des opportunités
d. Une personne qui supervise votre travail et vous donne du feedback sur vos performances

22. Laquelle de ces propositions permet d'optimiser la coordination entre les infirmières et les personnel médical ?

a. La capacité des infirmières à alerter les médecins sur le changement de conditions d'un patient
b. Une meilleure compréhension du raisonnement des médecins par les infirmières, qui conduit à améliorer la manière dont les infirmières exécutent les ordres
c. Réduire les conflits entre les professions en améliorant la communication
d. Toutes les options citées ci-dessus

23. Laquelle des propositions suivantes est un exemple de méthode d'évaluation par ses pairs ?

a. Comparer les résultats d'un professeur à un autre
b. Une notation différente de l'administrateur basée sur les résultats obtenus avec le patient
c. L'observation de notre technique d'enseignement par un autre professeur qui rend ensuite un feedback sur cette technique d'enseignement
d. Demander aux étudiants comment ils noteraient différents professeurs sur leur enseignement

24. Laquelle de ces phrases définit le mieux le professionalisme d'un professionnel de la santé ?
a. Prioritiser l'éducation d'un collègue avant tout
b. Prioritiser le remboursement majoré de votre profession lorsque c'est possible
c. Prioritiser la communication avec vos patients
d. Prioritiser les besoins de votre patient et de la société avant tout

25. Parmi les commentaires suivants, lequel est un commentaire d'évaluation?
a. Votre cours d'hier était bien organisé, mais vous devriez vous focaliser plus sur les maladies et moins sur les traitements
b. Vous avez bien travaillé lors de votre stage avec moi
c. J'ai remarqué que les pansements que vous mettiez sur les patients n'étaient pas bien attachés.
d. Vous êtes toujours à l'heure pour le travail, quelque chose que j'apprécie.

26. Parmi les méthodes suivantes, laquelle est la plus efficace pour enseigner en utilisant une présentation Powerpoint?
a. Mettre une photo sur une diapositive avec des mots résumant les points clé, et répéter ces points clé à l'oral.
b. Mettre une photo sur une diapositive et décrire oralement les concepts évoqués par la photo en utilisant un langage simple.
c. Mettre juste des mots sur une diapositive et parler des mots utilisés.
d. Mettre une photo sur une diapositive avec des mots résumant les points clé, puis décrire à l'oral les concepts évoqués par la photo.

27. Quelle est la première étape dans la définition d'un projet de recherche ?
a. Considérer les ressources disponibles pour l'accomplissement du projet
b. Vouloir répondre à une question spécifique
c. Effectuer une revue pertinente de la littérature
d. Soumettre des formulaires au comité d'examen institutionnel

28. Parmi les choses suivantes, laquelle est une "séquence agenda" plutôt qu'on objectif éducatif?
a. "Comprendre les différences clé entre épilepsie générale et partielle"
b. "Apprendre deux classifications différentes de l'anémie"
c. "Savoir utiliser l'ultrason pour diagnostiquer une ascite"
d. "Discuter la définition d'un accident cérébro-vasculaire"

29. Quelle doit-être la première étape dans l'élaboration d'un curriculum d'un nouveau programme de résidence?
a. Etablir des buts pour le curriculum.
b. Effectuer une étude générale pour comprendre les besoins du programme.
c. Identifier des stratégies éducatives adaptées au contenu du programme.
d. Etablir des dates et des heures pour tous les cours du programme.

Merci! Se il vous plaît passer le test de l'avant
